# Environmental cooperation system, ESG performance and corporate green innovation: Empirical evidence from China

**DOI:** 10.3389/fpsyg.2023.1096419

**Published:** 2023-03-02

**Authors:** Shi Qiang, Chen Gang, Huang Dawei

**Affiliations:** ^1^School of Management, Shenzhen Polytechnic, Shenzhen, China; ^2^School of Economics and Management, Harbin Institute of Technology Shenzhen, Shenzhen, China

**Keywords:** environmental cooperation system, corporate green innovation, ESG performance, difference-in-differences, corporate environmental social responsibility, executives’ overseas background

## Abstract

The Environmental Cooperation System (ECS) is a new exploration of the government’s spatial environmental policy to meet the requirements of green and sustainable development, so it is very important to scientifically evaluate its green innovation effect. Based on China’s A-share listed companies from 2006 to 2021, from the perspective of corporate ESG performance, we apply the multi-dimensional fixed-effects difference-in-differences (DID) model, and empirically test the impact, mechanism, and heterogeneity of the Environmental Cooperation System of Shenzhen-Dongguan-Huizhou Metropolis (ECS-SDHM) on corporate green innovation. It found that ECS-SDHM can significantly improve corporate green innovation, and the policy effect is more significant in the private enterprise group. Secondly, we use ESG rating score and decomposition indicators to deeply analyze the green innovation effect mechanism of ECS-SDHM from the perspective of ESG performance. The results show that ECS-SDHM can enhance corporate green innovation by significantly improving corporate ESG performance, environmental governance, and social governance. Further research found that both corporate environmental social responsibility and executives’ overseas backgrounds can positively moderate the green innovation effect of ECS-SDHM by positively moderating the ESG performance mechanism.

## 1. Introduction

Resource and environmental issues have become an important constraint restricting China’s economic growth, and the issue of green and sustainable development is imminent (Yu et al., 2020). The government needs to coordinate the relationship between economic development and environmental protection. In this context, green innovation has become a key driver of green sustainable development (Razzaq et al., 2021). How to realize the development of green innovation at the enterprise level has become an important topic of academic attention in recent years (Chen X. et al., 2018; Kraus et al., 2020).

Metropolitan circle planning is an important starting point for the Chinese government to stimulate regional economic growth and strengthen spatial governance. The Environmental Cooperation System in Metropolitan Circle (ECSMC) under the background of regional environmental collaborative governance is a new form of environmental spatial policy for the government to address environmental issues under the requirements of green and sustainable development. Existing theoretical and empirical literature on the influencing factors of corporate green innovation are abundant (Tang et al., 2020), but few scholars have carried out research on the impact of cross-regional spatial environment policies on corporate green innovation, and research on the impact of innovation is particularly lacking. To better solve the above problems, we choose Shenzhen-Dongguan-Huizhou Metropolis (SDHM), which has developed Environmental Cooperation System (ECS), as the research object among the many metropolitan circles in China. We construct policy dummy variables of the Environmental Cooperation System of Shenzhen-Dongguan-Huizhou Metropolis (ECS-SDHM), use various econometrics estimation methods such as multi-dimensional fixed-effects DID method to investigate the impact of China’s ECSMC on corporate green innovation.

Compared with developed economies, the foundation and driving force of developing economies to engage in green innovation activities are relatively weak, which requires the joint efforts of governments, enterprises, and third-party organizations to gradually achieve (Pedersen et al., 2021; Tan and Zhu, 2022). Environmental, Social and Governance (ESG) information disclosure can show investors the comprehensive corporate governance level from the three dimensions of corporate environmental governance, social governance, and corporate governance, which can make up for the deficiencies of traditional financial indicators, and provide investors with evaluation methods and indicators to further understand the overall picture of the company (Cohen et al., 2020; Tan and Zhu, 2022). Existing research has confirmed that better corporate ESG performance can attract more investors to enter the market, make positive market effects, greatly reduce the cost of capital for companies to engage in green innovation activities, and improve corporate green innovation performance (Wang et al., 2018; Cohen et al., 2020). At the same time, existing research shows that government intervention is an important driver of corporate environmental information disclosure, such as corporate ESG performance (Li et al., 2018; Yu et al., 2020). Therefore, the ESG performance of enterprises may be an important mechanism path for environmental policies to affect the green innovation of enterprises. However, the existing literature rarely discusses the impact mechanism of ECSMC on corporate green innovation from the perspective of corporate ESG performance. By manually collecting the financial characteristics data of listed companies from CSMAR database, and matching with the ESG index of Chinese listed companies disclosed by Bloomberg, we apply the fixed-effects panel DID model to examine the policy effect mechanism and the heterogeneity of policy effects from the perspective of corporate ESG performance.

In addition, we also try to examine its moderating effect on the green innovation effect of ECSMC from the perspectives of corporate social responsibility and overseas background of corporate executives. Combined with the mechanism of ESG, we further analyze the role of the moderating effect in the ESG mechanism.

The possible marginal contributions of this paper are: Firstly, we expand the research on the driving factors of enterprise green innovation from the perspective of ECSMC. Metropolitan circle’s environmental collaborative governance system is also an important part of environmental governance and will also affect corporate green innovation performance. However, the existing research literature on this is lacking. Secondly, this paper attempts to examine the mechanism path of ECSMC affecting green innovation of enterprises from the perspective of ESG performance. According to the external pressure theory, ECSMC is an important external environmental pressure, and the stakeholders of the enterprise will therefore exert sustainable governance pressure on the enterprise, and then improve the ESG performance of the enterprise. However, there is little literature on this process in existing studies. Thirdly, we further explore the moderating effect of corporate environmental social responsibility and executives’ overseas background on ECSMC’s impact on corporate green innovation. The above research provides ideas for follow-up scholars to introduce micro-effect assessments similar to ECSMC in other developing countries or regions.

The rest of this paper is organized as follows: Section 2 discusses the policy background and literature. Section 3 presents the research design. Section 4 presents the empirical results of the benchmark model, robustness tests, and ownership heterogeneity. Section 5 explores the mechanism of ESG. Section 6 shows the moderating effect analysis. The last section is the conclusions and limitations.

## 2. Background and literature

### 2.1. Policy background

The Pearl River Delta is an important urban agglomeration along the southeast coast of China. Shenzhen-Dongguan-Huizhou is the sub-center metropolitan circle in the multi-center circle structure of the Pearl River Delta urban agglomeration. In 2009, Shenzhen, Dongguan, and Huizhou established an environmental and ecological task force under the framework of the “Joint Meeting of Main Party and Government Leaders of the Three Cities.” On the one hand, it reflects the great importance attached by the three cities of Shenzhen, Dongguan and Huizhou to cross-city environmental protection cooperation, and on the other hand, it lays a clue for the subsequent ECS-SDHM establishment. In 2010, Shenzhen, Dongguan, and Huizhou formally established an environmental protection cooperation system among the three cities of Shenzhen, Dongguan, and Huizhou, and held the first cooperation meeting in the following year. As of 2022, the three cities of Shenzhen, Dongguan, and Huizhou have held more than 10 cooperation meetings, established environmental protection cross-inspection teams many times, and dispatched more than 400 inspectors to carry out environmental protection inspections on enterprises in their jurisdictions, focusing on atmospheric emission reduction and pollution control.

The goal of ECS-SDHM is to further promote SDHM environmental protection cooperation, increase joint cross-enforcement efforts, work together to prevent environmental risks, focus on solving cross-border environmental problems, and promote the high-quality economic development of the three cities with high-level protection of the regional ecological environment, and provide a strong guarantee for SDHM’s environmental security and social stability. In 2022, the Ministry of Ecology and Environment of China reports the national ambient air quality in February 2022. Among the 168 key cities, the air quality of SDHM’s 3 cities ranks among the top 20 in the country, among which Huizhou ranks 3rd, Shenzhen ranks 6th, and Dongguan ranks 19th. This shows that ECS-SDHM has made some progress in environmental governance. However, it is difficult for us to directly judge whether the establishment of ECS-SDHM has a positive impact on corporate green innovation. Issues such as its influencing mechanism and the heterogeneity of policy effects need to be further empirically analyzed through scientific policy identification methods.

### 2.2. Literature and hypothesis

#### 2.2.1. ECSMC and corporate green innovation

Firstly, ECSMC is obviously one of the environmental regulatory policies, and the conclusions of previous studies on the impact of environmental regulatory policies on corporate green innovation may also apply to ECSMC. Previous studies have shown that environmental regulatory policies have a significant impact on corporate green innovation (Amore and Bennedsen, 2016; Chen X. et al., 2018; Hazarika and Zhang, 2019; Takalo and Tooranloo, 2021). For example, Zhang D. et al. (2022) found that command-based environmental regulation can effectively promote enterprises’ green technology innovation ability. Liu et al. (2021) investigates the impact of China’s new Environmental Protection Law on the green innovation behavior of listed companies in high-polluting industries. Their research affirms the positive impact of environmental regulation policies on corporate green innovation. Therefore, logically, ECSMC may also have a significant impact on corporate green innovation.

Secondly, different from traditional local government environmental policies, ECSMC is a multi-regional environmental governance policy tool that crosses administrative boundaries. The main department of environmental governance is no longer just the local environmental management department, but all environmental governance departments of environmental protection cooperative cities are involved, focusing on the governance of cross-regional environmental problems. For example, when faced with multi-regional ECS, there will be “green barriers” in regional trade between enterprises, which may stimulate enterprises’ green supply chain innovation. It makes green logistics an effective way to strengthen the upstream and downstream links of inter -regional industrial chains, deal with regional “green barriers,” and achieve green sustainable development (Ren and Huang, 2015; Fan et al., 2022). It maybe an important path for ECSMC to promote corporate green innovation, that is, green supply chain innovation. Besides, in the context of ECSMC, listed companies will face more environmental policy enforcement and management and constraints from regulatory authorities. Therefore, when facing the same environmental problems, the environmental regulation intensity of listed companies has increased significantly. According to organizational legitimacy theory (Soewarno et al., 2019; Hamm et al., 2022) and stakeholder theory (Zhang and Zhu, 2019; Freeman et al., 2021), when the pressure of environmental legitimacy on enterprises rises significantly, the management of listed companies increases their decision-making motivation to engage in green innovation activities under the requirements of environmental legitimacy of stakeholders, thereby improving the level of green innovation of enterprises (Wang et al., 2020; Liu et al., 2021). Accordingly, we propose the first hypothesis:

*H_1_*: ECS-SDHM will significantly improve the green innovation level of local listed companies.

#### 2.2.2. Mechanistic role of ESG performance

An important contribution of this study is to try to examine the mechanism by which ECSMC affects corporate green innovation from the perspective of corporate ESG performance. Existing research has carried out a wealth of theoretical and empirical research on the relationship between ESG performance and corporate green innovation (Tolliver et al., 2021). These studies show that green innovation is a process of technological innovation to achieve the goals of resource conservation and environmental protection, and it has the characteristics of typical innovation activities with high risk, large investment and long return period. Enterprises in developing countries lack the motivation for active green innovation, and need the joint efforts of governments, social organizations, and enterprises. Under the policy constraints and market guidance, enterprises shift from passive governance to active green innovation (Eiadat et al., 2008; Pedersen et al., 2021; Tan and Zhu, 2022). ESG ratings play an important role in this process. The disclosure of corporate ESG information is a positive green signal to the market, which will attract more sustainable investors, ease investment and financing constraints and agency costs, reduce corporate risks, enhance green environmental decision-making motivation, and promote corporate green innovation (Tan and Zhu, 2022). For example, Tan and Zhu (2022) discussed the impact of ESG rating on green innovation of listed companies based on the characteristic data of Chinese A-share listed companies from 2010 to 2018 and the 2015 ESG rating of the SynTao Green Finance Agency. They found that ESG ratings significantly boosted the quantity and quality of corporate green innovation, and it was achieved by easing financial constraints and increasing managers’ environmental awareness.

Previous studies on the relationship between ECSMC and corporate ESG performance have been seldom covered. We try to understand how ECSMC affects corporate ESG performance from a theoretical perspective related to government intervention and corporate governance. Previous studies have shown that government environmental intervention (mainly environmental policy) is an important driver of corporate environmental information disclosure (Stephan, 2002; Shi et al., 2021). The decision-making of corporate environmental information disclosure is the release of green signals by corporate managers to stakeholders to meet the environmental legitimacy requirements of stakeholders in the context of environmental regulation. ECSMC is a multi-regional and cross-administrative environmental governance system, which exerts greater environmental regulatory pressure on enterprises in the jurisdiction. It forces companies to carry out positive green behavior decisions, and while improving corporate green performance, it will also significantly increase the incentives of listed company management to disclose environmental information, thereby obtaining better ESG rating performance. Accordingly, we propose the key mechanism hypothesis of the green innovation effect of ECSMC in this study:

*H_2_*: ECS-SDHM can significantly improve the ESG performance of local enterprises which leads to the improvement of corporate green innovation.

#### 2.2.3. Moderating role of environmental social responsibility and executives’ overseas background

According to the signal theory, when a listed company has a better attitude toward environmental and social responsibility or assumes more environmental and social responsibility, it can release green environmental protection signals to the market to ensure a certain advantage in the market-oriented investment environment, which will attract more investors’ attention and investment, reduce the cost of capital use of enterprises, and then stimulate green innovation activities and improve the level of green innovation of enterprises (Kraus et al., 2020; Shahzad et al., 2020). Accordingly, we propose the first hypothesis of moderating effect:

*H_3a_*: Enterprises’ environmental social responsibility has a positive moderating effect on ECSMC’s promotion of corporate green innovation.

Due to the different stages of economic development, China’s concept of green environmental protection lags behind developed countries, Which means that whether you have an overseas background may affect the content and direction of corporate decision makers’ green decisions. Previous studies have shown that corporate boards with senior executives monitor managerial decisions (Hussain et al., 2020), this makes whether senior executives have overseas experience a factor that may moderate the green innovation effect of ECS-SDHM. Ren and Hussain (2022) found a positive and significant effect of green human resource management (GHRM) on employee and firm environmental performance. Theoretically, if the senior management team of a listed company has overseas study and work experience, the overseas green cultural background and green economic behavior in the market economy will have a certain degree of influence on the cultural identity of the executives and the cognition of green economic decision-making. This cultural and cognitive impact will make them adopt overseas green economy cognition and behavior when they are faced with green environmental decisions after returning to China (Ren et al., 2021; Zhao et al., 2022), that is, they are more inclined to take positive green behaviors to respond to the government’s policy pressure and release positive green signals to the market, which attracts green investors, which in turn increases the motivation of green decision-making, such as green innovation decision-making, and enhances the green innovation level of listed companies (Fei et al., 2022). Accordingly, we propose the second hypothesis of moderating effect:

*H_3b_*: Corporate executives’ Overseas Background has a positive moderating effect on ECSMC’s promotion of corporate green innovation.

## 3. Research design

### 3.1. Methodology

To identify the policy effects of ECS-SDHM scientifically and credibly, we mainly use the multi-dimensional fixed-effects DID model to examine the impact of ECS-SDHM on green innovation of micro-enterprises. Accordingly, we construct the following multidimensional fixed-effects DID model to identify policy effects:


(1) $$\begin{array}{l} GAP_{ijt}=\beta \underset{DID_{it}}{\underset{︸}{Post_{t}\times Treat_{i}}}+\underset{k}{\sum }\rho_{k}X_{jk\left(t-1\right)}+Province_{j}\\ \;\;\;\;\;\;\;\;\;\;\;\;\;\;\;\;\;\;\;+Year_{t}+Province_{j}\;*Year_{t}+\varepsilon_{ijt} \end{array}$$

In the above model, *i* represents the listed company, *j* represents the province, and *t* represents the year. $GAP_{ijt}$ is the dependent variable, and represents the green innovation level of listed companies. $Post_{t}$ is the policy time dummy variable. $Treat_{i}$ is the dummy variable of the treatment group (whether the enterprise is within the spatial scope of the SDHM). $X_{jk\left(t-1\right)}$ is the control variable with a lag of one period. $Province_{j},Year_{t},Province_{j}*Year_{t}$ represent the province, time fixed effect items, and province*year interactive fixed effect term, respectively. $\varepsilon_{ijt}$ is the error term.

### 3.2. Variables

#### 3.2.1. Dependent Variable

Based on the research of Li and Zheng (2016), Wang and Wang (2021), etc., we use the number of green patent applications of listed companies to measure the level of corporate green innovation. In addition, in the follow-up research, we will conduct robustness test with the number of green invention patents and green utility model patents of listed companies as proxy variables of the dependent variable. Due to the obvious right-skewed distribution of the green patent data of listed companies, we adjusted the number of patents by adding 1 and then taking the natural logarithm to obtain the dependent variable $GAP_{ijt}$ of this study, and $GIAP_{ijt}$ and $GUAP_{ijt}$ in the robustness test. In addition, we use the ratio of the number of green patent applications filed by listed companies in the current year to the number of patents filed in the current year, $GAPRATIO_{ijt}$ , as another robustness test dependent variable.

#### 3.2.2. Key Explanatory Variable

The key explanatory variable in the benchmark model (1) is the multiplication term of the dummy variable of the time when the ECS-SDHM is established and the dummy variable of the treatment group whether the enterprise is within the spatial scope of the SDHM, that is, $Post_{t}$ is the ECS-SDHM time dummy variable, which is 0 before the policy is implemented and 1 after the policy is implemented. $Treat_{i}$ is a dummy variable of the treatment group, and the enterprise *i* is within the spatial range of the SDHM, and takes 1; otherwise, it takes 0. What we care about is the coefficient β of $Post_{t}\times Treat_{i}$ , if it is significantly greater than 0, it means that ECS-SDHM significantly improves corporate green innovation, and if it is less than 0, it means it significantly inhibits corporate green innovation.

#### 3.2.3. Key Mechanism Variables

The ESG disclosure performance data we use for listed companies comes from the Bloomberg database. The release of the index provides the possibility for the integration of corporate-level sustainability disclosures and simplified corporate ESG analysis (Zhang X. et al., 2022). In addition, we also use the ESG decomposition items provided by Bloomberg, namely corporate environmental governance, social governance, and corporate governance to investigate of which channel mechanism has a mechanism role in ECSMC’s impact on corporate green innovation. In addition, we also use the ESG index released by Shanghai China Securities Index as a surrogate indicator to test the robustness of the ESG mechanism.

#### 3.2.4. Control Variables

To control the potential confounding factors that may affect the green innovation of enterprises and obtain reliable policy effect estimation results, based on the research of scholars such as Zhang and Liu (2022), Si and Cao (2022), we control the following variables at the enterprise level: ① Enterprise size (Size); ② Asset-liability ratio (Lev); ③ Return on total assets (ROA); ④ Return on Equity (ROE); ⑤ Net asset turnover (ATO); ⑥ Enterprise TobinQ (TobinQ); ⑦ The age of the enterprise (Age); ⑧ Corporate cash flow ratio (CashFlow). In addition, we also control the fixed effect of the province where the enterprise is located, the fixed effect of year and the interactive fixed effect of province*year to remove the unobservable confounding factors at the interactive level of province, year and province-year, and improve the credibility of the policy effect estimation process.

### 3.3. Data sources

This paper manually collects the data of A-share listed companies from 2006 to 2021. Financial insurance and abnormal trading listed companies (ST and PT listed companies) are excluded, and samples of companies with serious missing variables are also removed. The data sources of this study mainly include three parts: One is the green patent data of listed companies. We obtain the green patent data of listed companies by matching the patent identification of listed companies in the China Research Data Service Platform (CNRDS) with the “Green List of International Patent Classification” issued by the World Intellectual Property Organization (WIPO). The second is the mechanism variable data. The corporate ESG performance data comes from the ESG disclosure scores of listed companies released by the Bloomberg database. The third is the control variable data. This part of the financial characteristics data of listed companies comes from the CSMRA database. Finally, we matched the listed company’s green patent data, ESG performance data, etc. with the listed company’s characteristic data, and finally got 39,873 observations. To eliminate the influence of extreme values, this paper conducts winsorize processing of up and down 1% for the main variables.

## 4. Empirical results

### 4.1. Parallel trend test

Based on the literature (Deschenes et al., 2017; Liu and Xiao, 2022), we use the event study method for reference to carry out the parallel trend test, and the model is as follows:


(2) $$\begin{array}{l} GAP_{ijt}=\overset{8}{\underset{m=-3}{\sum }}\lambda_{m}Treat_{i}\times Time_{m}+\underset{k}{\sum }\rho_{k}X_{jk\left(t-1\right)}+Province_{j}\\ \;\;\;\;\;\;\;\;\;\;\;\;\;\;\;\;\;\;\;+Year_{t}+Province_{j}*Year_{t}+\varepsilon_{ijt} \end{array}$$

**Figure 1:**
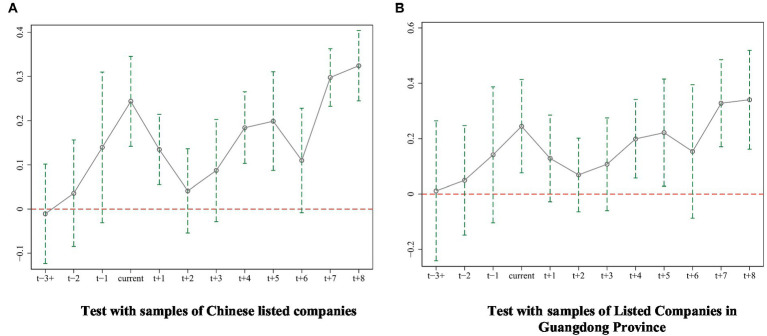
Parallel trend test. **(A)** Test with samples of Chinese listed companies and **(B)** Test with samples of listed companies in Guangdong Province.

In model (2), $Time_{m}$ is the year dummy variable. $\lambda_{m}$ is the coefficient of the difference between the treatment group and the control group affected by the policy effect in the corresponding year. We use the full sample and the Guangdong Province sample to estimate twice, and draw parallel trend test results according to the estimated results, as shown in Figures 1A,B. Obviously, there was no significant difference between the treatment group and the control group before the implementation of ECS-SDHM, which satisfied the parallel trend assumption.

### 4.2. Benchmark results

As shown in Table 1, columns (1)–(4) are average treatment effect results, respectively, estimated by the DID model with no control variable without fixed effect, without control variable with fixed effect, with control variable without fixed effect, and DID model including control variable and multidimensional fixed effect. Column (4) is the estimation result of the benchmark model (1). By comparison, we can examine the difference in estimated results with or without control variables and with or without fixed effects. The results show that, after controlling for enterprise-level control variables, province, year, and interactive fixed effects, ECS-SDHM shows a significant promoting effect on the green innovation of local listed companies, and the estimated coefficient is significant at the 1% confidence level. The above benchmark regression results confirm for the first time that China’s ECS-SDHM has significantly improved the green innovation of local enterprises, which is consistent with the theoretical hypothesis *H_1_*, and also confirms the relevant research conclusions that environmental policies promote green innovation of enterprises (Chen Z. et al., 2018; Hazarika and Zhang, 2019; Takalo and Tooranloo, 2021).

**Table 1:** Baseline regression results.

Model	(1)	(2)	(3)	(4)	(5)
*DID*	0.160***	0.257***	0.200***	0.203***	0.225***
	(0.029)	(0.029)	(0.000)	(0.007)	(0.041)
*Obs.*	15,339	13,869	15,318	13,849	2,324
R-squared	0.002	0.188	0.072	0.243	0.209
Controls	N	Y	N	Y	Y
Province*Year FE	N	N	Y	Y	N
Province FE	N	N	Y	Y	N
Year FE	N	N	Y	Y	Y

Ps: Robust standard errors in parentheses, ***p < 0.01.

In addition, column (5) in Table 1 is the estimation result of only selecting the listed companies in Guangdong Province to identify the benchmark model. It is not difficult to see that the benchmark estimation results are still significant at the 1% confidence level, which again supporting the theoretical hypothesis *H_1_*.

### 4.3. Robustness tests

#### 4.3.1. Placebo test

**Figure 2:**
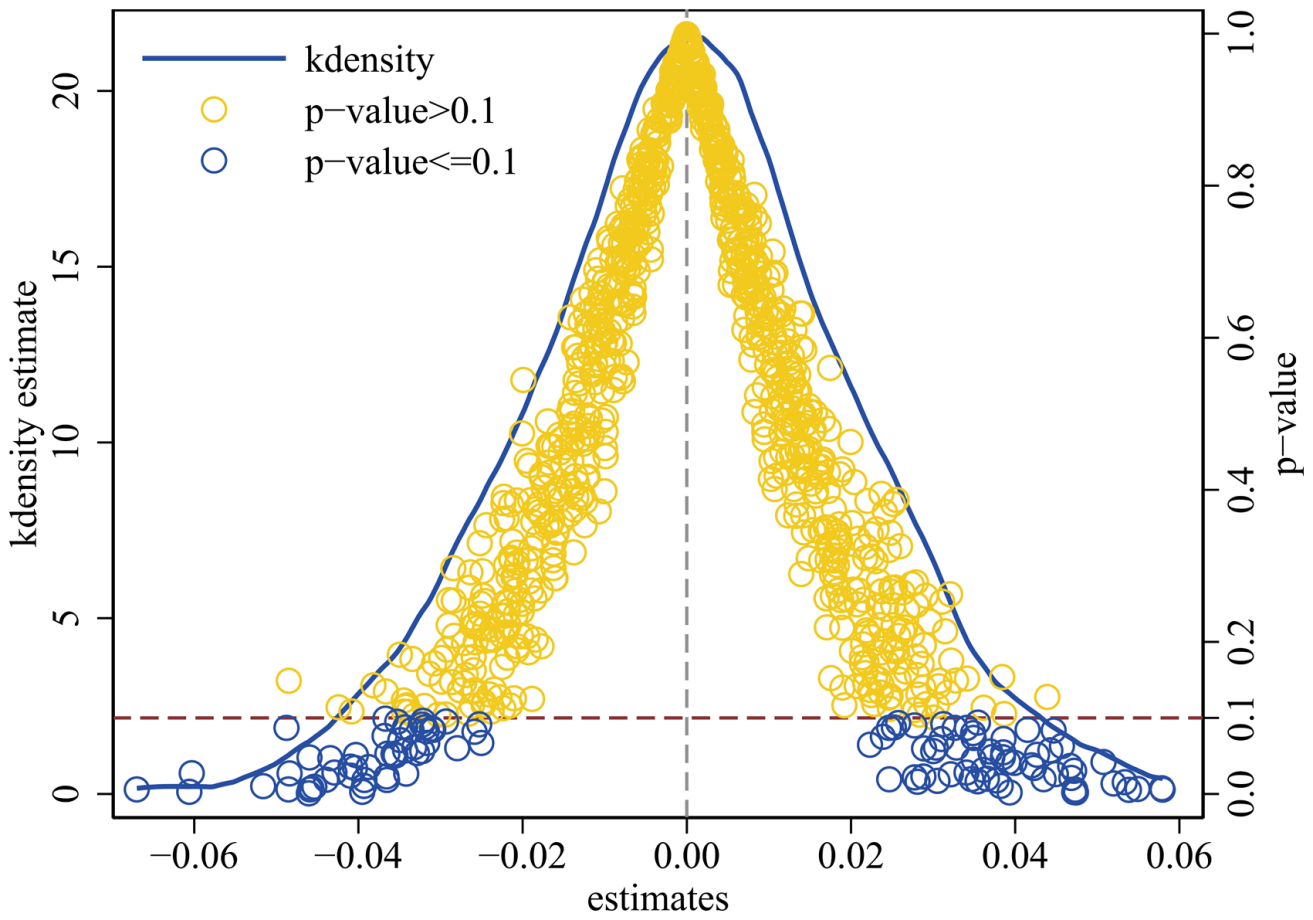
Placebo test.

Based on Huang and Chen (2022), Wang et al. (2021), we further avoid self-selection bias by conducting multiple random sampling experiments. Specifically, we run 1,000 random samplings to obtain a virtual treatment group and a control group, respectively apply the benchmark estimation model to identify policy effects, and obtain 1,000 estimation results. The results of the placebo test were examined by plotting the kernel density curve and the distribution of policy effect coefficients. Figure 2 shows the distribution of the DID coefficients in the random sampling estimation results. It can be found that most of the sampling estimation coefficients still fail to pass the hypothesis at the 10% confidence level, that is, the significance of the placebo sampling results basically fails, which indicates that the ECS-SDHM effect does not exist significantly in the random sampling simulation, that is, the baseline estimation results pass the placebo test.

#### 4.3.2. Change the dependent variable

Based on Xu and Cui (2020), we added three variables *GAPRATIO*, *GIAP* and *GUAP*, and re-estimates the benchmark model (1).

The estimation results are shown in Table 2. Columns (1), (3), and (5) in Table 3 are the analysis results of appling the benchmark model (1) to estimate policy effects when *GAPRATIO,* *GIAP,* and *GUAP* are used as dependent variables. It is not difficult to find that the estimated results of the three groups of dependent variables are all significantly positive at the 1% confidence level. It shows that under the corporate green innovation measured by various measurement methods, the promotion effect of ECS-SDHM on corporate green innovation is still significant, and the benchmark estimation results are robust, which verifies the hypothesis *H_1_* again.

**Table 2:** Baseline regression with different dependent variables.

Variables	*GAPRATIO*		*GIAP*		*GUAP*	
Model	(1)	(2)	(3)	(4)	(5)	(6)
*DID*	0.034***	0.035***	0.223***	0.244***	0.114***	0.129***
	(0.001)	(0.009)	(0.007)	(0.042)	(0.007)	(0.040)
Obs.	13,849	2,324	13,850	2,325	13,849	2,324
R-squared	0.072	0.046	0.222	0.211	0.217	0.172
Controls	Y	Y	Y	Y	Y	Y
Province*Year FE	Y	N	Y	N	Y	N
Province FE	Y	N	Y	N	Y	N
Year FE	Y	Y	Y	Y	Y	Y

Ps: Robust standard errors in parentheses, ***p < 0.01.

**Table 3:** Heckman two-step correction results.

Model	(1)	(2)	(3)	(4)	(5)	(6)
Variables	*ifGAP*	*GAP*	*ifGIAP*	*GIAP*	*ifGUAP*	*GUAP*
*DID*	0.177**	0.235***	0.246***	0.174***	0.155*	0.091***
	(0.081)	(0.049)	(0.081)	(0.062)	(0.082)	(0.027)
Obs.	34,619	13,637	34,425	10,804	34,413	10,432
R-squared		0.243		0.242		0.239
Controls	Y	Y	Y	Y	Y	Y
Province*Year FE	Y	Y	Y	Y	Y	Y
Province FE	Y	Y	Y	Y	Y	Y
Year FE	Y	Y	Y	Y	Y	Y

Ps: Robust standard errors in parentheses, ***p < 0.01, **p < 0.05, *p < 0.1.

In addition, we also estimate the sample from Guangdong Province. Columns (2), (4), and (6) are the analysis results with GAPRATIO, GIAP, and GUAP as dependent variables, respectively. It is not difficult to see that when the sample is limited to Guangdong Province, the estimated results are still significant at the 1% confidence level, again supporting the theoretical hypothesis *H_1_*.

#### 4.3.3. Heckman two-step correction

This paper discusses the influence of ECS-SDHM on the level of green innovation of enterprises. The estimated sample of the benchmark model is the sample with GAP>0, that is, the sample of listed companies whose number of green patents applied for in the current year is greater than 0. However, the sample with *GAP* = 0 is removed from the estimation, which may lead to sample selection bias in the policy effect estimation result. To eliminate the sample selection bias that may be caused by deleting *GAP* = 0 samples, we further use the Heckman two-step method (Heckman, 1976; Yang and Ma, 2022) model for robustness testing:


(3) $$\begin{array}{c} \begin{array}{l} Step\;1:if\;GAP_{ijt}=\;\;\;\;\upsilon DID_{it}+\underset{l}{\sum }\sigma_{l}Z_{jl\left(t-1\right)}+Province_{j}+Year_{t}\\ \;\;\;\;\;\;\;\;\;\;\;\;\;\;\;\;\;\;\;\;\;\;\;\;\;\;\;\;\;\;\;\;\;\;\;\;\;\;\;\;\;\;+Province_{j}*Year_{t}+\varepsilon_{ijt} \end{array}\\ \begin{array}{l} Step\;2\;GAP_{ijt}=\;\;\;\beta \underset{DID_{it}}{\underset{︸}{Post_{t}\times Treat_{i}}}+\underset{k}{\sum }\rho_{k}X_{jk\left(t-1\right)}+\zeta IMR_{it}\\ \;\;\;\;\;\;\;\;\;\;\;\;\;\;\;\;\;\;\;\;\;\;\;\;\;\;\;\;\;\;\;\;\;\;\;\;\;+Province_{j}+Year_{t}+Province_{j}*Year_{t}+\varepsilon_{ijt} \end{array} \end{array}$$

In the formula, $ifGAP_{ijt}$ is the dummy variable of whether $GAP_{ijt}$ is 0, that is, the binary dummy variable of whether the listed company chooses green innovation. $IMR_{it}$ is the inverse Mills ratio estimated by the probit model in Step 1, which is used to control the sample self-selection problem. In theory, the estimated $IMR_{it}$ in Step 1 contains unobservable information in sample selection We bring the $IMR_{it}$ estimated in Step1 into Step2 to correct the sample selection bias that may exist in the benchmark model.

The estimated results are shown in Table 3. The results show that the baseline model estimation results are still significant after considering the sample selection bias, and after using GIAP and GUAP as dependent variables to conduct robustness test using Heckman two-step model, the results are still significant, that is, after controlling for the sample selection bias, the theoretical hypothesis *H_1_* is supported again.

#### 4.3.4. Control the confounding effects of other policies

To further exclude other confounding effects that may have on ECSMC’s corporate green innovation effect, such as the Guangdong-Hong Kong-Macao Greater Bay Area (Wu et al., 2021), green credit policy (Hu et al., 2021), environmental law policy (Fang et al., 2021), based on the research of Liu and Xiao (2022), this paper constructs policy dummy variables of Guangdong-Hong Kong-Macao Greater Bay Area policy, green credit policy and environmental protection law policy, respectively. The Guangdong-Hong Kong-Macao Greater Bay Area policy and SDHM have overlapping processing groups, so there is an interaction effect, which is controlled by introducing interaction terms. The dummy variables of green credit policy and environmental protection law policy are directly incorporated into the benchmark model to control confounding effects.

The estimated results are shown in Table 4. Columns (1) ~ (3) are the estimated results of controlling the confounding effects of the Guangdong-Hong Kong-Macao Greater Bay Area policy, green credit policy and environmental protection law, respectively. The DID estimation results are all significantly positive at the 1% level, which shows that after controlling the confounding effects of the three policies, ECS-SDHM still has a positive effect on the green innovation of local enterprises. The hypothesis *H_1_* is verified again, and the robustness of the benchmark estimation results is verified.

**Table 4:** Control the confounding effects estimation result.

Model	(1)	(2)	(3)
Variables	YGA	Green credit	Environmental law
*DID*	0.249***	0.188***	0.202***
	(0.008)	(0.006)	(0.006)
*DDD*	−0.237***		
	(0.003)		
*yga*	0.349***		
	(0.010)		
*GnCrd*		0.325***	
		(0.023)	
*EnvLaw*			0.232***
			(0.021)
Obs.	13,849	13,869	13,869
R-squared	0.244	0.218	0.218
Controls	Y	Y	Y
Province*Year FE	Y	N	N
Province FE	Y	Y	Y
Year FE	Y	N	N

Ps: Robust standard errors in parentheses, ***p < 0.01.

### 4.4. Ownership heterogeneity

We believe that state-owned enterprises and private enterprises may enjoy different “treatment” when facing government policies. Therefore, we analyze ownership as an important source of heterogeneity for our estimation analysis. Based on the practice of Jiang (2022), we apply a DDD estimation model to investigate whether ECS-SDHM will have a heterogeneous green innovation promotion effect for different types of listed company entities. This model can not only directly show the difference of the policy effect coefficient between the state-owned and private groups, but also can be achieved by testing *H_SOE_*: $\gamma_{1}=\gamma_{2}$ . The test model is as follows:


(4) $$\begin{array}{l} \begin{array}{c} GAP_{ijt}\\ GAPRATIO_{ijt},GIAP_{ijt},GUAP_{ijt} \end{array}=\;\;\;\;\;\;\;\begin{array}{c} \;\gamma_{1}DID_{it}\times SOE_{it}+\gamma_{2}DID_{it}\\ \times \;\;\left(1-SOE_{it}\right)+\theta SOE_{it} \end{array}\\ \;\;\;\;\;\;\;\;\;\;\;\;\;\;\;\;\;\;\;\;\;\;\;\;\;\;\;\;\;\;\;\;\;\;\;\;\;\;\;\;\;\;\;\;\;\;\;\;\;\;\;\;\;\;\;\;\;\;\;\;\;\;\;\;\;\;\;\;\;\;\;\;\;\;\;\;\;\;\;\;\;\;+\underset{k}{\sum }\rho_{k}X_{jk\left(t-1\right)}+Province_{j}\\ \;\;\;\;\;\;\;\;\;\;\;\;\;\;\;\;\;\;\;\;\;\;\;\;\;\;\;\;\;\;\;\;\;\;\;\;\;\;\;\;\;\;\;\;\;\;\;\;\;\;\;\;\;\;\;\;\;\;\;\;\;\;\;\;\;\;\;\;\;\;\;\;\;\;\;\;\;\;\;\;\;\;\;+Year_{t}+Province_{j}*Year_{t}+\varepsilon_{ijt} \end{array}$$

The estimated results are shown in Table 5. Columns (1) ~ (4) are the estimation results under different dependent variables. The results show that the policy effect coefficients of state-owned listed companies are smaller than those of private listed companies under the four dependent variables, and the *H_SOE_*: $\gamma_{1}=\gamma_{2}$ tests of *GAP*, *GIAP* and *GUAP* all reject the null hypothesis. It shows that the private listed companies in metropolitan circle get more green innovation promotion effect under ECS-SDHM, and the coefficient difference is significant. It is inconsistent with the traditional perception that state-owned enterprises may be taken care of by policies, and private enterprises are not subject to significant “policy discrimination” in ECSMC. The possible explanation is that ECS-SDHM is a government-mandated and restrictive policy, which is different from the welfare policy issued by the government, and private enterprises are often subject to greater policy pressure in the face of restrictive policies. Therefore, under greater policy pressure, private corporate stakeholders tend to be more inclined to make green decisions, thereby enhancing the level of corporate green innovation. State-owned listed companies are often subject to less policy pressure than private enterprises under restrictive policies. Therefore, the pressure transferred to the stakeholders of listed companies is obviously weaker than that of private listed companies, and then the policy effect is weaker than that of private units. This explanation is also more in line with China’s national conditions.

**Table 5:** Ownership heterogeneity estimation results.

Model	(1)	(2)	(3)	(4)
Variables	*GAP*	*GAPRATIO*	*GIAP*	*GUAP*
DID*SOE	0.029	0.032***	0.086**	−0.086
	(0.036)	(0.004)	(0.032)	(0.075)
DID*(1*-SOE*)	0.237***	0.038***	0.254***	0.148***
	(0.014)	(0.002)	(0.014)	(0.023)
Obs.	13,849	23,680	13,850	13,849
R-squared	0.244	0.056	0.222	0.219
Controls	Y	Y	Y	Y
Province*Year FE	Y	Y	Y	Y
Province FE	Y	Y	Y	Y
Year FE	Y	Y	Y	Y
F(*H_SOE_*)	19.02	0.96	14.45	11.02
*value of p*(*H_SOE_*)	0.000***	0.336	0.001***	0.001**

Ps: Robust standard errors in parentheses, ***p < 0.01, **p < 0.05.

## 5. Mechanism analysis

We use the ESG performance scores of Chinese listed companies published by Bloomberg as the dependent variable (ESG) for the mechanism test, and use the ESG performance scores of Chinese listed companies published by the Shanghai China Securities Index as a surrogate variable (ESGHZ) for robustness testing. Accordingly, this paper constructs the following model to test the hypothesis *H_2_*:


(5) $$\begin{array}{l} \underset{ESGHZ_{ijt}}{ESG_{ijt}}=\beta \underset{DID_{it}}{\underset{︸}{Post_{t}\times Treat_{i}}}+\underset{k}{\sum }\rho_{k}X_{jk\left(t-1\right)}+Province_{j}\\ \;\;\;\;\;\;\;\;\;\;\;\;\;\;\;\;\;\;\;\;\;\;\;+Year_{t}+Province_{j}*Year_{t}+\varepsilon_{ijt}\; \end{array}$$

The estimated results are shown in columns (1) and (3) of Table 6. It found that the results of column (1) show that when uses Bloomberg publishes ESG score to measure the ESG performance of listed companies, ECS-SDHM has a positive effect on the ESG performance of listed companies, and the estimated coefficient is significant at the 1% level. It shows that ECS-SDHM significantly improves the ESG performance level of local enterprises. The results in column (3) show that when the ESGHZ of the China Securities Index is used to measure the ESG performance of listed companies, ECS-SDHM still has a positive effect on the ESG performance of listed companies, and the estimated coefficient is significant at the 5% level. It has repeatedly demonstrated that improving the level of ESG performance is an important mechanism for ECS-SDHM to improve the level of corporate green innovation, that is, the hypothesis *H_2_* is proved.

**Table 6:** ESG mechanism estimation results.

Variables	ESG		ESGHZ	
Model	(1)	(2)	(3)	(4)
*DID*	0.027***		0.002**	
	(0.005)		(0.001)	
DID*SOE		0.031*		0.018***
		(0.017)		(0.005)
DID*(1*-SOE*)		0.033***		0.007**
		(0.008)		(0.003)
Obs.	5,660	5,660	13,076	13,076
R-squared	0.328	0.330	0.210	0.234
Controls	Y	Y	Y	Y
Province*Year FE	Y	Y	Y	Y
Province FE	Y	Y	Y	Y
Year FE	Y	Y	Y	Y
F(*H_SOE_*)		0.00		1.94
*value of p*(*H_SOE_*)		0.957		0.174

Ps: Robust standard errors in parentheses, ***p < 0.01, **p < 0.05, *p < 0.1.

Secondly, to examine whether there is a difference in corporate ownership in the ESG performance mechanism, we further test based on model (4). Columns (2) and (4) in Table 6 are the estimated results of the ownership heterogeneity of the ESG performance improvement effect. Our study found that the estimated coefficients of DID*SOE and DID*(1*-SOE*) were both significantly positive, but the *H_SOE_* test showed that there was no significant difference between the two coefficients. It shows that the improvement effect of ECS-SDHM on corporate ESG performance does not show significant differences in ownership.

In addition, we also use the ESG decomposition indicators provided by Bloomberg, namely corporate environmental governance (Environ), social governance (Social), and corporate governance (Govern) as the mechanism variable, investigate of which channel mechanism in the ESG mechanism plays a role in the green innovation effect of ECSMC. Accordingly, we construct the following model for testing:


(6) $$\begin{array}{l} \underset{Social_{ijt},Govern_{ijt}}{Environ_{ijt}}=\beta \underset{DID_{it}}{\underset{︸}{Post_{t}\times Treat_{i}}}+\underset{k}{\sum }\rho_{k}X_{jk\left(t-1\right)}+Province_{j}\\ \;\;\;\;\;\;\;\;\;\;\;\;\;\;\;\;\;\;\;\;\;\;\;\;\;\;\;\;\;\;\;\;\;\;\;\;+Year_{t}+Province_{j}*Year_{t}+\varepsilon_{ijt} \end{array}$$

The estimated results are shown in columns (1), (3) and (5) of Table 7. It found that ECS-SDHM had a significant positive effect on the environmental governance and social governance of listed companies in the jurisdiction, and the estimated coefficients were all significant at the 1% level. However, it does not have a significant impact on the corporate governance of listed companies. This result shows that the promotion effect of ECS-SDHM on the ESG performance of listed companies in the jurisdiction is mainly achieved by improving the level of environmental governance and social governance of enterprises.

**Table 7:** ESG decomposition indicators mechanism estimation results.

Variables	Environ		Social		Govern	
Model	(1)	(2)	(3)	(4)	(5)	(6)
*DID*	0.024***		0.054***		0.001	
	(0.008)		(0.006)		(0.002)	
DID*SOE		−0.002		0.103***		−0.013***
		(0.029)		(0.021)		(0.004)
DID*(1*-SOE*)		0.041**		0.044***		0.009***
		(0.019)		(0.014)		(0.003)
Obs.	4,991	4,991	5,569	5,569	5,660	5,660
R-squared	0.246	0.246	0.217	0.219	0.253	0.258
Controls	Y	Y	Y	Y	Y	Y
Province*Year FE	Y	Y	Y	Y	Y	Y
Province FE	Y	Y	Y	Y	Y	Y
Year FE	Y	Y	Y	Y	Y	Y
F(*H_SOE_*)		0.93		3.44		11.14
*value of p*(*H_SOE_*)		0.343		0.073*		0.002***

Ps: Robust standard errors in parentheses, ***p < 0.01, **p < 0.05, *p < 0.1.

Secondly, to examine the ownership differences in the ESG performance decomposition indicator mechanism, we further test it based on model (4). Columns (2), (4), and (6) in Table 7 are the estimation results under different ESG decomposition indicators. We found that the estimated coefficient of DID*SOE of the listed company’s environmental governance level mechanism is negative, and the estimated coefficient of DID*(1*-SOE*) is positive, but the coefficient difference is not significant by *H_SOE_* test. It shows that the promotion effect of ECS-SDHM on the environmental governance level of listed companies is more reflected in the private enterprise group, and the state-owned enterprise does not show a significant promotion effect. The estimated coefficients of DID*SOE and DID*(1*-SOE*) of social governance are both significantly positive and there are significant differences. It shows that from the perspective of social governance, the improvement effect of ECS-SDHM on the social governance level of state-owned listed companies is greater than that of private listed companies. This result affirms that Chinese state-owned enterprises show the main characteristics in undertaking social responsibility, which is consistent with the social governance responsibility goal in the government’s management goal of state-owned enterprises. In addition, the estimated coefficient of DID*SOE of corporate governance is negative, and the estimated coefficient of DID*(1*-SOE*) is positive, and the two coefficients are significantly different. It shows that although ECS-SDHM does not show a promotion effect on the corporate governance level of listed companies at the overall sample level, ECS-SDHM has played a policy effect from the perspective of ownership grouping. However, the policy effect is only a facilitation effect in private enterprises, and a depressing effect in state-owned enterprises. The possible explanation for this result is that compared with private enterprises, state-owned enterprises need to pay more attention to social governance given by the state, which will inevitably have a certain crowding effect on corporate governance, that is, sacrificing some corporate governance goals to pay more meet social governance goals.

## 6. Further analysis

### 6.1. Moderating effect of environmental social responsibility (ESR)

Based on the measurement of environmental and social responsibility of listed companies by Si and Cao (2022), we use the front-end governance (FG), end-end governance (EG) and employee green behavior (EGB) of listed companies to comprehensively measure listed companies’ environmental and social responsibility. To investigate and verify the moderating effect of ESR, we constructed the DDD estimation model (7), which can identify the coefficient differences by testing *H_ESR_*: $\gamma_{1}=\gamma_{2}$ is as follows:


(7) $$\begin{array}{l} GAP_{ijt}=\gamma_{1}DID_{it}\times \begin{array}{c} FG_{it}\\ EG_{it},EGB_{it} \end{array}+\gamma_{2}DID_{it}\times \left(1-\begin{array}{c} FG_{it}\\ EG_{it},EGB_{it} \end{array}\right)\\ \;\;\;\;\;\;\;\;\;\;\;\;\;\;\;\;\;\;\;+\theta \begin{array}{c} FG_{it}\\ EG_{it},EGB_{it} \end{array}\;+\underset{k}{\sum }\rho_{k}X_{jk\left(t-1\right)}+Province_{j}+Year_{t}\\ \;\;\;\;\;\;\;\;\;\;\;\;\;\;\;\;\;\;\;\;+Province_{j}*Year_{t}+\varepsilon_{ijt} \end{array}$$

Columns (1) to (3) in Table 8 are the estimation results of the moderating effects of front-end governance (FG), end-end governance (EG) and employee green behavior (EGB) on the green innovation effect of ECS-SDHM. The two interaction coefficients of the three environmental social responsibilities are significant at the 1% level, and the *H_ESR_* test results of columns (1) and (3) are significant. It shows that there is a significant difference in the coefficients of interaction terms, and the difference is that the coefficients of interaction terms with environmental social responsibility are significantly larger than those without environmental social responsibility. It means that environmental social responsibility has a significant moderating effect on the green innovation effect of ECS-SDHM, and it is a positive moderating effect, which verifies hypothesis *H*_*3a*_. Specifically, the green innovation effect of ECS-SDHM is positively moderated through two forms of environmental and social responsibility, front-end governance and green behavior of employees.

**Table 8:** Moderating effect of ESR estimation results.

Model	(1)	(2)	(3)
DID*FG	0.374***		
	(0.097)		
DID*(*1-FG*)	0.168***		
	(0.009)		
DID*EG		0.860***	
		(0.084)	
DID*(*1-EG*)		0.185***	
		(0.010)	
DID*EGB			0.355***
			(0.070)
DID*(*1-EGB*)			0.176***
			(0.008)
Obs.	30,503	30,503	30,503
R-squared	0.237	0.229	0.228
Controls	Y	Y	Y
Province*Year FE	Y	Y	Y
Province FE	Y	Y	Y
Year FE	Y	Y	Y
F(*H_ESR_*)	19.20	1.15	9.55
*value of p*(*H_ESR_*)	0.000***	0.293	0.010**

Ps: Robust standard errors in parentheses, ***p < 0.01, **p < 0.05.

We cannot help but wonder whether this moderating effect plays a role in the ESG performance mechanism? Theoretically, if a listed company has a better attitude toward environmental and social responsibility or assumes more environmental and social responsibility, the company will tend to actively adopt positive green environmental behaviors. In turn, the company performs better in environmental, social and corporate governance, and gets better ESG performance scores from third-party assessment agencies. This releases a positive green investment signal to the capital market, attracts better ESG investment, stimulates green environmental decision-making behavior, improves the level of green innovation, and forms a positive feedback process (Gillan et al., 2021; Karwowski and Raulinajtys-Grzybek, 2021). Accordingly, we take ESG performance as the dependent variable and use the DDD model (7) to test, and the estimation results are shown in Table 9.

**Table 9:** Moderating effect of ESR on ESG estimation results.

Model	(1)	(2)	(3)
DID*FG	0.038***		
	(0.012)		
DID*(*1-FG*)	−0.034***		
	(0.006)		
DID*EG		−0.009	
		(0.019)	
DID*(*1-EG*)		−0.009***	
		(0.003)	
DID*EGB			0.042***
			(0.014)
DID*(*1-EGB*)			−0.034***
			(0.004)
Obs.	9,559	9,559	9,559
R-squared	0.332	0.285	0.301
Controls	Y	Y	Y
Province*Year FE	Y	Y	Y
Province FE	Y	Y	Y
Year FE	Y	Y	Y
F(*H_ESR_*)	16.84	0.00	18.98
*value of p*(*H_ESR_*)	0.000***	0.972	0.000**

Ps: Robust standard errors in parentheses, ***p < 0.01, **p < 0.05.

Columns (1) to (3) in Table 9 are the test and estimation results of the moderating effects of front-end governance (*FG*), end-end governance (*EG*) and employee green behavior (*EGB*) on ESG performance mechanism, respectively. Except for DID*EG, the two interaction coefficients of the three environmental social responsibilities are all significant at the 1% level, and the *H_ESR_* test results of columns (1) and (3) are significant. It shows that there is a significant difference in the interaction coefficient between “with or without front-end governance and employee green behavior” and DID. This difference is manifested in the fact that the coefficient of interaction of front-end governance and employees’ green behavior is significantly larger than that of no front-end governance and employees’ green behavior, that is, the two environmental social responsibilities of front-end governance and employees’ green behavior have a significant positive moderating effect on the ESG performance mechanism, which is basically consistent with the theoretical analysis. However, the interaction term of end-point governance did not show significant coefficient differences, that is, the environmental social responsibility did not show a significant moderating effect.

### 6.2. Moderating effect of executives’ overseas background

We construct a DDD estimation model (8) to test the moderating effect of executives’ overseas background, and identify the coefficient differences by testing *H_OSEA_*: $\gamma_{1}=\gamma_{2}$ . The specific model is as follows:


(8) $$\begin{array}{l} GAP_{ijt}=\gamma_{1}DID_{it}\times Oversea_{it}+\gamma_{2}DID_{it}\times \left(1-Oversea_{it}\right)+\theta Oversea_{it}\\ \;\;\;\;\;\;\;\;\;\;\;\;\;\;\;\;\;\;+\underset{k}{\sum }\rho_{k}X_{jk\left(t-1\right)}+Province_{j}+Year_{t}+Province_{j}*Year_{t}+\varepsilon_{ijt} \end{array}$$

Columns (1) to (4) in Table 10 are the model estimation results under different dependent variables. The estimated results of DID*Oversea and DID*(1*-Oversea)* were all significantly positive at the 1% level, and the test results of *H_OSEA_* were significant when *GAP*, *GIAP*, and *GUAP* were dependent variables. It shows that there is a significant difference between the estimated coefficients of DID*Oversea and DID*(1-*Oversea*), and the difference is that the coefficient of the interaction term of DID**Oversea is significantly larger than that of DID*(1-*Oversea*), that is, the overseas study and work background of the senior management team has a significant positive moderating effect on the green innovation effect of ECS-SDHM, which verifies hypothesis *H*_*3b*_.

**Table 10:** Moderating effect of executives’ overseas background estimation results.

Model	(1)	(2)	(3)	(4)
Variables	*GAP*	*GAPRATIO*	*GIAP*	*GUAP*
DID*Oversea	0.239***	0.038***	0.208***	0.143***
	(0.014)	(0.002)	(0.026)	(0.026)
DID*(1*-Oversea*)	0.113***	0.034***	0.099***	0.069***
	(0.018)	(0.003)	(0.023)	(0.018)
Obs.	28,173	22,435	28,174	28,173
R-squared	0.224	0.054	0.212	0.199
Controls	Y	Y	Y	Y
Province*Year FE	Y	Y	Y	Y
Province FE	Y	Y	Y	Y
Year FE	Y	Y	Y	Y
F(*H_OSEA_*)	17.46	0.92	10.09	6.84
*value of p*(*H_OSEA_*)	0.000***	0.344	0.009***	0.024**

Ps: Robust standard errors in parentheses, ***p < 0.01, **p < 0.05.

In addition, from the perspective of corporate governance, an important prerequisite for the performance of ESG disclosure to stimulate green innovation of enterprises is the recognition and support of senior managers of the enterprise for the economic value behind ESG disclosure. Existing research shows that corporate senior managers with overseas study and work backgrounds are more likely to accept the concept of green development, and they tend to take positive green behaviors when faced with environmental policy pressures, which in turn have a more positive impact on ESG information disclosure. Therefore, theoretically, the overseas background of executives will have a moderating effect on the impact of corporate ESG on corporate green innovation. Accordingly, we take ESG performance and three decomposition indicators as dependent variables, and use the DDD model (11) for testing. The estimated results are shown in Table 11.

**Table 11:** Moderating effect of executives’ overseas background on ESG estimation results.

Model	(1)	(2)	(3)	(4)
Variables	ESG	Environ	Social	Govern
DID*Oversea	0.054***	0.118***	0.091***	0.012*
	(0.014)	(0.039)	(0.018)	(0.006)
DID*(1*-Oversea*)	0.018***	0.033*	0.047***	−0.002
	(0.004)	(0.018)	(0.006)	(0.002)
Obs.	5,660	4,989	5,569	5,660
R-squared	0.330	0.341	0.217	0.253
Controls	Y	Y	Y	Y
Province*Year FE	Y	Y	Y	Y
Province FE	Y	Y	Y	Y
Year FE	Y	Y	Y	Y
F(*H_OSEA_*)	7.15	4.74	5.38	5.69
*value of p*(*H_OSEA_*)	0.012**	0.038**	0.027**	0.024**

Ps: Robust standard errors in parentheses, ***p < 0.01, **p < 0.05.

Columns (1) to (4) in Table 11 are the estimated results using model (8) when ESG, Environ, Social and Govern are the dependent variables, respectively. The estimation results of DID*Oversea and DID*(1-*Oversea*) under the four dependent variables are all significant (except the DID*(1-*Oversea*) coefficient under Govern is not significant), and the test results of *H_OSEA_* are all significant. It shows that the estimated coefficients of DID*Oversea and DID*(1-*Oversea*) are significantly different in the four estimation results. This difference shows that the DID*Oversea interaction term coefficient is significantly larger than the DID*(1-*Oversea*) coefficient, that is, the overseas study and work background of the executive team has a significant positive moderating effect on the ESG performance mechanism. This is basically consistent with the theoretical analysis, and this adjustment mechanism exists and is significant in environmental governance, social governance and corporate governance.

## 7. Conclusion and limitations

ECSMC promotes the high-quality economic development of metropolitan areas through regional ecological and environmental cooperation, which is an important exploration of cross-regional environmental governance. The scientific evaluation of ECSMC’s micro-green innovation effect is an important basis for the exploration of such green and sustainable policies. For the first time, based on the quasi-natural experiment of ECS-SDHM in China, our study examines the policy effect of ECSMC on corporate green innovation, and provides new perspectives and new ideas for the research on the influencing factors of corporate green innovation. Empirical research applying the multi-dimensional fixed-effects DID model found that: Firstly, ECS-SDHM can significantly improve corporate green innovation, and the policy effect has a more significant role in promoting green innovation among private listed companies, which is consistent with the conclusion of the green innovation effect of traditional environmental policies (Shen et al., 2020; Zhang T. et al., 2022). Secondly, from the perspective of corporate ESG performance, we found that the ESG performance is an important mechanism for ECS-SDHM to improve corporate green innovation, and ECS-SDHM is mainly achieved by improving the performance of environmental governance and social governance in the performance of corporate ESG. However, few existing studies have explored the policy effect of ECSMC on corporate green innovation from this perspective. Thirdly, corporate environmental and social responsibility and executives’ overseas study and work background can positively moderate the green innovation effect of ECS-SDHM through the positive moderating of the ESG performance mechanism. In general, China’s ECS-SDHM has achieved the goal of corporate green innovation effect. The above research provides ideas for subsequent scholars to introduce micro-effect evaluation similar to ECSMC in other developing countries or regions.

In addition, based on the above research, we try to make the following recommendations to policy-making institutions: Firstly, the government should pay more attention to ECSMC and expand the field of regional environmental cooperation. Try to institutionalize the successful measures of ECSMC and promote them to other metropolitan areas to expand the green innovation effect of ECS. Secondly, strengthen corporate ESG information disclosure, broaden ESG information disclosure channels, and enhance the scientific nature of ESG assessment. Introduce more companies to participate in ESG information disclosure, no longer limited to listed companies. Thirdly, increase the publicity of corporate environmental and social responsibilities, and broaden the channels for the introduction of overseas green talents.

Although from the perspective of corporate ESG performance, this paper provides an empirical test from SDHM in China for the corporate green innovation effect of ECSMC, however, it is subject to several limitations: ①In terms of research objects, only the ECS of SDHM in China is the research object, and there is a lack of discussion on other Metropolitan Circles in China and ECSMC in other developing countries; ②In terms of research perspective, although this paper examines the micro-policy effect mechanism path of ECSMC from the perspective of corporate ESG performance. However, the analysis and robustness test are only carried out from the ESG disclosure data of two third parties, and there is no in-depth comparison of the possible confounding effects of different third-party entities on corporate ESG evaluation and disclosure. Future research can try to expand the research scope and objects, deeply explore the potential impact of ESG evaluation subjects, and apply more advanced quantitative identification methods to examine the impact, mechanism and heterogeneity of ECSMC on corporate green innovation. Future research can consider the following ideas for improvement: ①Expand the research samples, such as China’s Guangdong-Hong Kong-Macao Greater Bay Area environmental protection integration system, etc., to broaden the limitations caused by the research samples; ②Attempt to use the independence of ESG disclosure institutions as a potential influencing factor to further investigate the ESG performance mechanism for causal identification, so as to reduce the confounding effect of the heterogeneity of ESG disclosure entities on the research conclusions.

## Data availability statement

The data analyzed in this study is subject to the following licenses/restrictions: The data are not publicly available due to privacy. Requests to access these datasets should be directed to HD, hdw@szpt.edu.cn.

## Author contributions

SQ, CG, and HD: conceptualization, validation, formal analysis, data curation, writing—original draft preparation, writing—review and editing, visualization, and supervision. CG and SQ: methodology. CG and HD: project administration and funding acquisition. All authors contributed to the article and approved the submitted version.

## Funding

We acknowledge the financial support from Natural Science Foundation of Guangdong Province 2023 Project (No. 2023A1515011868), Special project of dual-region research of Guangdong Province (Project No. GD22SQYJ02) and Shenzhen Polytechnic Fund (Project No. 6022310016S). Sponsored by Guangdong Modern Industry & SME Development Research Center.

## Conflict of interest

The authors declare that the research was conducted in the absence of any commercial or financial relationships that could be construed as a potential conflict of interest.

## Publisher’s note

All claims expressed in this article are solely those of the authors and do not necessarily represent those of their affiliated organizations, or those of the publisher, the editors and the reviewers. Any product that may be evaluated in this article, or claim that may be made by its manufacturer, is not guaranteed or endorsed by the publisher.
